# Cholera

**Published:** 1910-09-24

**Authors:** 


### SPECIAL ARTICLE.

### CHOLERA.

### ITS SYMPTOMATOLOGY AND DIFFERENTIAL DIAGNOSIS.

The occurrence in Russia, Germany, and elsewhere in Europe of epidemic and sporadic cases of cholera during the last few months brings home to one the fact that cases may occur at home, not only at seaport towns, but also inland, when sailors from infected ships have landed and gone on visits to friends. Sporadic cases may at any time be the starting-point of epidemics which, once established, may be difficult to eradicate. Early diagnosis and isolation of the source of infection are the essentials from the public health point of view. There is considerable variation in the symptoms of cholera, both in different epidemics and in different individual cases. The ordinary type of the disease exhibits certain well-marked stages, which have been designated the stage of incubation, the stage of evacuation, the stage of collapse, and the stage of reaction. The malady is due, as is now well enough established, to a virulent comma-shaped bacillus, which is actively motile and is known as the cholera vibrio.

Incubation.

The *stage of incubation* is that which intervenes between reception of the poison and the manifestation of serious disturbances of health; its duration varies from a few hours to a few days, seldom exceeding ten, generally averaging from three to six. There may be slight symptoms even during this period, though this is generally not the case; if any are present they take the form of transient diarrhœa, with a feeling of oppression at the pit of the stomach, and these are accompanied by certain general symptoms, of which the most important are malaise, pallor, headache, and depression of spirits.

The *stage of evacuation* generally comes on very acutely, and lasts from two to twelve hours, the duration depending partly on the dosage of the microbe and partly upon the resisting powers of the patient. The strongest-looking individuals are sometimes the worst affected. Purging is the main symptom, the motions being frequent, copious, and watery. The earlier stools are fæculent, but they rapidly become pale and relatively free from food residues, until they resemble water in which rice has been boiled; rice-water stools of this type are characteristic either of cholera or of acute arsenical poisoning. On standing, a flocculent or curdy sediment comes down. Later motions contain more or less food. There may be no abdominal pain at all, but occasionally there is severe griping of a colicky nature. Purging precedes vomiting, but the latter occurs sooner or later, and it may be noted that food which has been some time in the stomach shows little or no effects of digestion. Emesis is rapid, with much violence, and the vomited fluid becomes tinged with blood. When the stomach has become entirely empty, extremely painful retching may continue as in extreme cases of sea-sickness. Muscular cramps of a very painful nature are a prominent feature of the case at this time; they are most marked in the legs, in the abdomen, and back, and in some cases the whole muscular system may be affected.

The Final Stage.

There may or may not have been pyrexia to begin with, but as the stage of evacuation persists the patient's temperature becomes sub-normal, the skin becomes cold, pale or dusky, and covered, especially as to the extremities, hands, and forehead, with a cold clammy sweat; the features resemble those of the so-called facies Hippocratica with drawn cheeks, sunken eyes, pinched lips and nose, and a generally anxious expression; there is great thirst, much restlessness, and the pulse is increased in frequency and diminished in force without the rate of breathing being much affected. This is the *stage of collapse*, and it is very apt to have a fatal result. Gastro-intestinal symptoms may continue, but the most alarming signs are those of extreme exhaustion. The pulse may become hardly perceptible, beating at the rate of 120 to 140 or more at the wrist, the first heart-sound becomes weak and short, and the diastole and systole are of equal length. The surface of the body becomes livid from deficiency in the capillary circulation; breathing becomes shallower and laboured, the intelligence, hitherto clear, becomes clouded, the voice weak and feeble, thirst extreme, the urine suppressed, the temperature (whether in the mouth or axillæ) reduced to 97° F., 95° F., or in fatal cases possibly down to 90° F., even rectal temperatures being decidedly subnormal. The motions are at this time devoid of smell unless they become retained; both the breath and the skin exude a peculiar and characteristic odour. Death occurs from sheer exhaustion, generally preceded by coma, unless the *stage of reaction* is reached; if so, there is a slow but steady progression in the strength of the pulse at the wrist; hardly palpable for a time, it becomes more steady and more full. The coldness, lividity, clamminess, and the pinched appearance of the skin give rise to warmth and greater fulness. The body temperature rises to normal or above normal, the motions become less frequent and less watery, and from being like rice-water they pass through deepening tints of grey and brown until they become of normal colour. Vomiting ceases and small quantities of fluid food are retained; the urine, which has up till now been entirely suppressed, reappears after some hours: at first scanty, highly coloured, of strong smell, high specific gravity, and containing indican, albumin, and renal tube casts, it progressively becomes more watery and more abundant, until finally it is restored entirely to normal. Within a few days in a favourable case convalescence is established, though the condition is by no means free from complications and sequelæ.

### Atypical Cases.

The above being what may be termed a characteristic attack of cholera, it is important to know that other types of the disease are by no means uncommon. Thus there is the *ambulatory* form, in which there is little more than slight malaise, anorexia, and transient diarrhœa, rapidly passing off and attracting little notice unless such a case occurs in the midst of a general epidemic. The diagnosis depends, of course, upon examination of the stools for the characteristic cholera vibrio or comma bacillus, and seeing that ambulatory cases may be a source from which a virulent epidemic may arise, they should be borne in mind, and the stools, if need be, investigated bacteriologically. Next in degree come cases in which there is definite diarrhœa with or without vomiting, and sometimes associated with cramps, the attack lasting, however, only twenty-four hours or thereabouts, and ending in comparatively speedy recovery without suppression of urine and without any stage of collapse. The disease has arrested itself apparently in the stage of evacuation. Other cases, again, omit the evacuation stage altogether, a fatal result coming about without either vomiting or purging. Fortunately this form of the disease, which has been called *cholera sicca*, is very rare, for it is exceedingly difficult to diagnose without post-mortem examination.

### Complications and Sequelæ.

As regards complications and sequelæ the most important are imperfect reaction with a continuance of the symptoms of the stage of evacuation until the patient sinks into the typhoid stage and ultimately dies of exhaustion. Relapse is by no means uncommon, and it may be induced by the use of purgatives or by indiscretions in diet. Hyperpyrexia, especially when the thermometr reading is taken per rectum, is a very fatal sign, particularly when it develops during the stage of collapse. The rectal temperature may reach 109° F., that at the axilla being 107° F. at the same time. Skin eruptions, particularly urticaria, erythema, and less often roseola, maculæ, and bullæ, have all been described as part of the disease. Suppression of the urine with associated renal changes not infrequently leads to acute uræmia, and until the reappearance of urine has taken place the case is one of continued anxiety. Stupor with restlessness, momentary delirium, spasmodic twitching of muscles, bloodshot eyes, contracted pupils, dried lips and tongue, sordes, vomiting, and slow pulse are the chief symptoms of this choleraic uræmia, fatal coma being very liable to supervene, and jaundice, if it should occur, is a very fatal symptom.

Of the sequelæ of cholera one would mention in particular anæmia, nervous depression, debility, gastric irritability, persistent headache, insomnia, impairment of the intellect, general anasarca from renal changes, chronic diarrhœa, and, in pregnant women, abortion. Inflammatory lesions, particularly of the lungs, are also to be feared; bronchitis, pleurisy, œdema of the lungs or pneumonia, meningitis, arthritis, and parotitis have all been described. There is also a great tendency to sloughing of the skin, so that bedsores are not infrequent, ulceration of the cornea is to be guarded against, and gangrene of the nose, ears, penis, scrotum, and, more rarely, of the toes or fingers may lead to general septic infection and death.

### Post-mortem Findings.

Post-mortem the most characteristic lesions are to be found in the digestive tract, particularly in the small intestine near to the ileo-cæcal valve. The stomach is generally empty, its mucosa congested, with variable numbers of submucous ecchymoses, but seldom hæmorrhage into the cavity. The duodenum and jejunum usually exhibit hyperæmia, either patchy or continuous. The mucous membrane of the small intestine is generally swollen, sodden, and acutely hyperæmic, and a pulpy material consisting mostly of granular cells and of amorphous exudate can be scraped off it; there may even be superficial excoriation or ulceration. These changes become more and more marked as one descends the small bowel. The large intestine is generally less affected than the small. The peritoneal coat of the bowel has a rosy hue, though there is no general peritonitis; the mesenteric glands are acutely enlarged and hyperæmic.

The intestinal discharges and contents exhibit the following constituents miscroscopically: (1) The débris of food; (2) the result of epithelial infiltration and glandular irritation in the form of amorphous granular and protoplasmic masses, and granular catarrhal cells; the amount of epithelial cells found in the intestinal contents after death exceeds that discovered in the stools during life; (3) red blood corpuscles and leucocytes; (4) micro-organisms, some peculiar to cholera, others incidental.

The diagnosis of the disease from other gastro-intestinal affections which might simulate it depends upon the discovery of the cholera vibrio in the dejecta on cultural or direct bacteriological examination.

